# Prevention of intravascular catheter-related infections—25 years later

**DOI:** 10.1017/ice.2025.10300

**Published:** 2026-01

**Authors:** Leonard A. Mermel, Niccolò Buetti

**Affiliations:** 1 Division of Infectious Diseases, Department of Medicine, Warren Alpert Medical School of Brown University and Department of Epidemiology and Infection Prevention, Brown University Health, Providence, RI, USA; 2 Infection Control Program and World Health Organization Collaborating Centre, Faculty of Medicine, Geneva University Hospitals, Geneva, Switzerland; 3 INSERM, IAME, Université Paris-Cité, Paris, France

There was no financial support for this manuscript. Dr. Mermel has served on a scientific advisory committee to Citius Pharma, he was a consultant for Pristine Access Technologies, and he serves as a subject matter expert for the AHRQ Safety Program for HAI Prevention. Dr. Buetti has no conflicts of interest related to this manuscript.

Due to the widespread use of intravascular catheters in hospitals around the world, infections related to these devices remain problematic. These infections predominantly arise from microbes colonizing the catheter insertion site or catheter hub.^1^ This Letter to the Editor aims to assess preventative strategies that have arisen over the last quarter century to reduce the risk of these infections and remaining unmet needs. Additionally, we highlight prevention strategies that were not included in the 2022 Society for Healthcare Epidemiology of America (SHEA)/Infectiuos Diseases Society of America (IDSA)/Association for Professionals in Infection Control and Epidemiology (APIC) Compendium practice recommendations for CLABSI prevention^2^ based on more recent data in the peer-reviewed literature. These recommendations are based on expert opinion. What has changed since this topic was extensively reviewed in 2000^3^ regarding prevention of catheter-related bloodstream infections (CRBSIs) are listed in the Table. Some of these changes are as follows: the subclavian vein is now the preferred site of central venous catheter insertion for reducing risk of CRBSI; alcoholic chlorhexidine has become a standard of care to cleanse the insertion site; chlorhexidine-containing dressings have become a standard of care at the insertion site of central venous catheters; and chlorhexidine bathing of intensive care unit (ICU) patients is highly recommended in several countries.^2^ Although antimicrobial coated or impregnated central venous catheters are recommended if CRBSI rates are high, it is important to realize that studies supporting their efficacy are decades old when many preventative strategies noted in the Table were not standards of care. Thus, it is unclear if the use of antimicrobial central venous catheters is as impactful today in mitigating risk of CRBSI in light of these other interventions and further studies are needed to assess their efficacy in the current era. If there is a high risk of infections associated with long-term catheters despite basic infection prevention measures (eg, patients requiring long-term catheterization who have a history of recurrent CRBSIs), then use of an antimicrobial lock solution as prophylaxis is recommended.^2^ Alcohol-containing barrier caps have been demonstrated to reduce risk of CRBSI and are recommended if basic preventative measures have not afforded institutions to reach their CRBSI target.^4^ Use of a device incorporating chlorhexidine as part of an end cap with an intraluminal extension has been demonstrated to reduce risk of CRBSI involving hemodialysis catheters.^5^ As such, their use is recommended. A promising intervention involves electively replacing non-tunneled short-term catheters in the internal jugular vein and femoral vein if *in situ* for greater than 10–14 days and continued central venous access remains necessary.^6^ However, adoption of this practice awaits future clinical trials with outcome measures including infectious outcomes and noninfectious complications that may occur with central venous catheter (CVC) insertion.

Some of the above-noted recommendations also noted in the Table were not included in the 2022 Compendium.^3^ Consideration for changing internal jugular and femoral vein CVCs is based on a study published after the Compendium was published.^6^ The support for this consideration also reflects findings of an elegant, if not forgotten, study which found that “patients whose CVC remains in situ for 5 days or less to have a 1% chance of acquiring a BSI, by day 5. However, physicians should expect the risk of BSI to increase to 6% at day 15 and to 21% by day 30”.^7^ Thus, this data, along with others, reveal risk of CRBSI follows a rising but non-uniform curve over time. At the very least, clinical teams should be aware of this rising risk carefully weighed against the necessity for continued catheterization in each patient. Use of prophylactic antimicrobial lock solutions for patients with long-term CVCs at high risk of CRBSI is based on a systematic review of data in meta-analyses which demonstrated reduced risk of CRBSI in this patient population.^8^ This too was published after the 2022 Compendium. As such, prophylactic lock use should be considered in patients requiring long-term CVC use, particularly if they have a history of CRBSI and/or limited alternative venous access.

The review published in 2000 and the 2022 Compendium did not focus on short-term peripheral venous catheters (PVCs), midline catheters, or peripherally-inserted central catheters (PICCs). Approximately 330 million PVCs are purchased yearly in the US alone and such widespread use leads to substantial risk of bloodstream infection.^9^ This risk of bloodstream infection can be reduced by routinely replacing PVCs every 3–4 days in adult patients.^10^

Myriad preventative strategies have reduced CRBSI risk but there remains an ongoing tension regarding behavior change and harnessing technologic advances to further reduce risk. Integrated continuous quality improvement efforts are essential but with the “getting to zero” mindset of risk reduction, technologic advances are also imperative. Although many novel catheters, lock solutions and catheter hubs have been investigated,^11,12^ few have come into widespread use. The cost to develop these technologies, particularly those that do not add to the risk of antimicrobial resistance, performing randomized trials, and eventual up front cost to healthcare institutions remain barriers to moving novel technologies into clinical practice.

Many challenges in the prevention of CRBSIs remain unresolved. Most current surveillance systems do not include short-term PVC-related bloodstream infections, nor infections associated with midline catheters. Further efforts are needed to measure the risk posed by these devices and to assess the impact of infection prevention strategies. This may occur when the forthcoming National Healthcare Safety Network hospital-onset bacteremia and fungemia measure becomes available for hospital-based surveillance programs. Moreover, in the era of digital health and informatics, the role of automation and artificial intelligence in BSI surveillance requires further definition and development. Ideally, novel surveillance strategies should be integrated with innovative interventional approaches, such as adaptive trial platforms, which allow the evaluation of multiple preventive interventions within a single, flexible framework. In sum, prevention of BSIs associated with intravascular devices has advanced over the last quarter century. Yet, the battle continues to marshal all of the available resources to further drive down risk to the patients we serve.

**Table 1.** Recommendations for the prevention of intravascular catheter-related infection

	Author’s recommendations in 2000^3^	Author’s recommendations in 2025
Catheter insertion		
Subcutaneous tunneling short-term CVCs inserted in the internal jugular or femoral veins when CVCs are not used for drawing blood	Yes	No
Full barrier precautions during CVC insertion	Yes	Yes
Contamination shield for pulmonary artery catheters	Yes	Yes
Preparation of insertion site with CHG-containing antiseptics	Yes	Yes, use alcoholic CHG
Prophylaxis with vancomycin and other therapeutic agents	No	No
Femoral vein catheter insertion	No	No
Subclavian vein rather than internal jugular vein CVC insertion	Yes	Yes
Preparation of insertion site with tincture of iodine	Yes	Yes, if alcoholic CHG unavailable
Full barrier precautions during insertion of midline, peripheral artery, and pulmonary artery catheters	Yes	Yes
Catheter maintenance		
Routine replacement of CVCs	No	Consider replace in 10–14 d*°
Chlorhexidine-silver sulfadiazine–impregnated short-term CVCs	Yes	Yes, if CRBSI rate high
Low-dose heparin for patients with short-term CVCs	Yes	No
Specialized nursing teams caring for patients with short-term peripheral venous catheters at institutions with a high incidence of infection	Yes	Yes
Povidone-iodine ointment applied to hemodialysis catheter insertion sites	Yes	No
Hub with chamber filled with iodinated alcohol for CVC with an expected duration of approximately 2 weeks	Yes	No, use alcohol-containing end cap
Povidone-iodine–saturated sponge enclosed in plastic casing fitted around the hubs of central venous catheters with an expected 2-week duration	Yes	No
Minocycline-rifampin–impregnated short-term CVCs	Yes	Yes, if CRBSI rate high
Triple antibiotic ointments applied to insertion sites	No	No
Silver-impregnated subcutaneous collagen– cuffed short-term CVCs	No	No
Silver-impregnated subcutaneous collagen– cuffed long-term CVCs	No	No
Low-dose warfarin for patients with long-term CVCs	Yes	No, use thrice weekly recombinant TPA for hemodialysis catheters
Transparent or gauze dressing for CVCs	Yes	Yes
Gauze dressings when blood is oozing from the insertion site	Yes	Yes
Adequate nurse-to-patient ratio in ICUs	Yes	Yes
Change needleless system, device, and end cap (if present) on a regular basis in accordance with the manufacturer’s guidelines and reduce contact with nonsterile water	Yes	Yes
Continuing quality improvement programs to improve compliance with catheter care guidelines	Yes	Yes
Remove catheters as soon as possible after intended use	Yes	Yes
Disinfect catheter hubs and sampling ports before accessing	Yes	Yes
Pulmonary artery catheters heparin-bonded with benzalkonium chloride	Yes	Yes
Povidone-iodine ointment applied to insertion sites non-tunneled CVCs of immunocompromised patients with heavy Staphylococcus aureus carriage	Yes	No, attempt decolonization
Specialized nursing teams caring for patients with catheters used for total parenteral nutrition	Yes	Yes
Hub with chamber filled with iodinated alcohol or hub-protective povidone-iodine–saturated sponge for heavily manipulated CVCs in ICUs	Yes	No, use alcohol-containing end cap
Mupirocin ointment applied to the insertion site	No	No
Inline filters	No	No
Prophylactic antimicrobial lock solution for patients with long-term CVCs at high risk of CRBSI^†^	N/A	Yes°
Change short-term peripheral venous catheters at 3-4 d intervals in adult patients^†^	N/A	Yes

PICC, peripherally-inserted central catheter; CVC, central venous catheter; ICU, = intensive care unit; TPA, tissue plasminogen activator; CHG, chlorhexidine; CRBSI, catheter-related bloodstream infection

*For femoral or internal jugular CVC; ^†^These were not included in the 2000 publication; °These recommendations were not included in the 2022 Compendium regarding CVC insertion and maintenance
